# Microbiota and Pancreatic Cancer: New Therapeutic Frontiers Between Engineered Microbes, Metabolites and Non-Bacterial Components

**DOI:** 10.3390/cancers17223618

**Published:** 2025-11-10

**Authors:** Sara Sofia De Lucia, Enrico Celestino Nista, Marcello Candelli, Sebastiano Archilei, Franziska Deutschbein, Enrico Capuano, Antonio Gasbarrini, Francesco Franceschi, Giulia Pignataro

**Affiliations:** 1 Department of Medical and Surgery Sciences, Università Cattolica del Sacro Cuore di Roma, 00168 Roma, Italy; sarasofia.delucia@guest.policlinicogemelli.it (S.S.D.L.); enricocelestino.nista@policlinicogemelli.it (E.C.N.); marcello.candelli@policlinicogemelli.it (M.C.); sebastiano.archilei01@icatt.it (S.A.); franziska.deutschblein01@icatt.it (F.D.); enrico.capuano01@icatt.it (E.C.); antonio.gasbarrini@policlinicogemelli.it (A.G.);; 2 Department of Emergency, Anesthesiologic and Reanimation Sciences, Fondazione Policlinico Universitario Agostino Gemelli IRCCS, 00168 Rome, Italy

**Keywords:** pancreatic cancer, pancreatic adenocarcinoma, microbiota, probiotic, postbiotic, microbiota transplantation

## Abstract

**Simple Summary:**

Pancreatic ductal adenocarcinoma is one of the deadliest forms of cancer, with survival rates that have changed little over time. Traditional treatments are often ineffective, highlighting the urgent need for new strategies. Recent studies have shown that microorganisms living in the body (such as bacteria, fungi, and viruses) can influence the development and response of pancreatic cancer to therapy. This review examines the impact of these microbial communities on tumor growth, the immune system, and the efficacy of current treatments. It also examines innovative therapeutic approaches that use beneficial microbes or target harmful ones, including probiotics, engineered bacteria, bacteriophages, and fecal microbiota transplantation. With a better understanding and manipulation of the relationship between the microbiome and cancer, researchers may discover new ways to diagnose, prevent, and treat pancreatic cancer more effectively.

**Abstract:**

Pancreatic ductal adenocarcinoma (PDAC) remains one of the most aggressive and lethal human malignancies, with five-year survival rates showing only marginal improvement despite decades of intensive research. Its dismal prognosis reflects a combination of intrinsic biological aggressiveness, late clinical presentation, and marked resistance to standard therapies, underscoring the urgent need for innovative diagnostic and therapeutic approaches. Growing evidence indicates that the microbiome is a modifiable factor influencing the onset, progression, and treatment response of PDAC. Microbial communities originating from the gut, oral cavity, and even the tumor microenvironment can shape carcinogenic pathways, modulate immune activity, and alter the efficacy of chemotherapy and immunotherapy. In addition to bacteria, fungal and viral populations are emerging as relevant contributors within this complex ecosystem. This review provides a comprehensive overview of the current mechanistic and translational evidence linking the microbiome to PDAC biology and therapy. It further explores microbiota-targeted interventions—such as probiotics, postbiotics, engineered bacterial strains, bacteriophages, oncolytic viruses, and fecal microbiota transplantation—as promising adjuncts to conventional treatments. A deeper understanding of host–microbiome interactions could yield novel biomarkers and open innovative avenues for precision medicine in PDAC, ultimately improving patient outcomes and reshaping therapeutic paradigms. Integrating microbiome-based strategies into PDAC management may thus represent a crucial step toward more effective and personalized oncologic care.

## 1. Introduction

The human gut microbiota constitutes a highly diverse ecological community of bacteria, fungi, viruses, and parasites, collectively comprising approximately 100 trillion microorganisms that play a pivotal role in maintaining host health [1]. Once considered a passive colonizer, the microbiota is a dynamic regulator of host physiology, orchestrating immune maturation, nutrient and drug metabolism, and the maintenance of mucosal integrity [2,3]. Disruptions in microbial composition or diversity have been associated with a wide range of pathological conditions, including malignancies [4,5]. The concept of bidirectional communication between the gut and distant organs is well established, with the gut–brain axis serving as the classical model [6]. More recently, this paradigm has been expanded to include a gut–pancreas axis, highlighting the reciprocal interactions between these systems. Pancreatic secretions—comprising digestive enzymes and antimicrobial peptides—can influence intestinal microbial communities, whereas microbial dysbiosis and subsequent translocation across the gut barrier may trigger pancreatic inflammation [7]. This bidirectional relationship is particularly relevant in the context of pancreatic ductal adenocarcinoma (PDAC), the most prevalent pancreatic malignancy and the sixth leading cause of cancer-related mortality worldwide [8,9]. Despite significant advances in oncology, the prognosis for PDAC remains poor, with five-year survival rates showing minimal improvement due to late-stage presentation and the absence of effective screening strategies [8]. Established risk factors include cigarette smoking, chronic pancreatitis, diabetes mellitus, family history, alcohol consumption, obesity, and inherited predisposition [10]. However, these factors alone cannot fully explain the onset of disease, suggesting the involvement of additional contributors. Among these, the microbiome has emerged as a potential key determinant [10]. Increasing evidence implicates microbial communities, including those from the gut, oral cavity, and intratumoral niches, in the initiation and progression of PDAC [10]. Dysbiosis has been linked not only to carcinogenesis but also to the remodeling of the tumor microenvironment, thereby modulating immune regulation and therapeutic efficacy [11]. Intratumoral microorganisms, in particular, may affect responsiveness to chemotherapy and immunotherapy, opening the door to precision-based therapeutic approaches [12]. This review synthesizes current evidence on the intricate interplay between the microbiota and pancreatic cancer, with a focus on dysbiosis, microbial metabolites, and the emerging role of non-bacterial components. It further explores how host–microbe interactions influence the tumor microenvironment and therapeutic response and discusses innovative microbiome-centered strategies—including microbiota editing and engineered microbial consortia—as potential supplements to existing therapies.

## 2. Materials and Methods

Relevant publications were identified through comprehensive searches of the PubMed, Scopus, and Embase databases. The search strategy combined Medical Subject Headings (MeSH) terms and free-text keywords, including “pancreatic cancer,” “pancreatic ductal adenocarcinoma,” “microbiota,” “gut microbiota,” “dysbiosis,” “oral microbiota,” “periodontal disease,” “fecal microbiota transplantation,” “probiotics,” “prebiotics,” “antibiotics,” and “microbial metabolites.” Boolean operators (“AND,” “OR”) were used to refine or broaden the search appropriately. The search was limited to peer-reviewed English-language articles published within the past 20 years. Following the removal of duplicates, all retrieved records were screened for relevance. Titles and abstracts were assessed for eligibility, with full-text review conducted when preliminary screening suggested potential inclusion. Studies were eligible if they examined any aspect of the relationship between the microbiota (intestinal, oral, or other body sites) and pancreatic ductal adenocarcinoma (PDAC), including mechanistic insights, diagnostic or prognostic implications, and therapeutic relevance. Both original research and review articles were included to ensure a broad and balanced synthesis of available evidence. The methodological quality and scientific rigor of each study were evaluated, with particular attention to study design, sample size, analytical techniques, and relevance to the research objectives. Data from eligible studies was narratively synthesized to identify convergent findings, clarify discrepancies and current knowledge gaps, and outline emerging trends in microbiota–PDAC research.

## 3. Oncobiosis and Microbial Metabolite Signaling in Pancreatic Adenocarcinoma

### 3.1. Oral and Oral–Gut Microbiota Axis in the Pathogenesis and Progression of Pancreatic Adenocarcinoma

The oral cavity hosts a complex microbial ecosystem comprising thousands of microorganisms collectively known as the oral microbiota [13]. Increasing evidence indicates that these microbial species are essential for maintaining oral homeostasis but may also contribute to oncogenic processes, particularly under conditions of dysbiosis [14]. Oral health is closely tied to the composition of this microbiome; for example, periodontal disease—an inflammatory condition marked by immune dysregulation—can result from alterations in the oral microbial community [15]. Oral dysbiosis has been associated with a variety of systemic diseases, including diabetes, atherosclerosis, and cancer [16]. Multiple lines of evidence suggest that periodontal pathogens are linked to the development of several malignancies, including pancreatic cancer [17]. Notably, periodontal disease is now recognized as an independent risk factor for pancreatic cancer [18]. In a prospective study, elevated serum antibodies against *Porphyromonas gingivalis* (*P*. *gingivalis*) ATCC 53978 were detected years before diagnosis. They were significantly more prevalent among individuals who later developed pancreatic cancer, suggesting that oral dysbiosis may predispose to this malignancy even up to five years prior to its onset [19]. Moreover, *P. gingivalis* has been identified in pancreatic lesions in murine models and shown to induce pancreatic acinar-to-ductal metaplasia in vitro [20]. In addition, the dynamic interplay between oral and gut microbial communities—referred to as the “oral–gut microbiota axis”—has been shown to contribute to the pathogenesis of pancreatic ductal adenocarcinoma (PDAC) through digestive and circulatory translocation toward the pancreatic gland [21]. Finally, a recent review by Papa et al. reported that several oral species, including *Neisseria elongata, Streptococcus mitis, Granulicatella adiacens,* and *Aggregatibacter actinomycetemcomitans*, are associated with an increased risk of pancreatic cancer [22]. Although the underlying pathogenic mechanisms remain incompletely elucidated, these findings open promising avenues for early diagnosis and risk stratification, as well as for the development of innovative microbiome-based therapeutic strategies.

### 3.2. Role of the Gut Microbiota in the Pathogenesis and Progression of Pancreatic Adenocarcinoma

The gut microbiota is increasingly recognized as a key factor in pancreatic tumorigenesis and disease progression. In a prospective study, Nagata et al. analyzed fecal samples from patients with pancreatic cancer and compared them with those from healthy controls [23]. They reported an enrichment of *Veillonella spp*. and *Actinomyces spp*., along with a reduction in Clostridiales—including unclassified Lachnospiraceae, *Eubacterium ventriosum*, and *Faecalibacterium prausnitzii*—particularly among individuals with advanced disease [23]. A subsequent metagenomic shotgun sequencing study examined the association between single-nucleotide polymorphisms (SNPs) within gut bacterial genomes and pancreatic cancer. Among the analyzed taxa, Lachnospiraceae exhibited the highest SNP density, particularly in patients with tumors located in the pancreatic head, where SNP abundance was inversely correlated with pancreatic cancer risk [24]. The role of *Helicobacter pylori* (*H. pylori*) in pancreatic carcinogenesis remains a topic of controversy. While a recent study found no association between *H. pylori* seropositivity and pancreatic cancer risk [25], earlier reports suggested a positive link [26]. Thus, the contribution of *H. pylori* to pancreatic tumorigenesis remains unresolved. Beyond bacteria, fungal communities may also participate in pancreatic oncogenesis. In particular, *Malassezia* species have been identified as more abundant in the mycobiome of both murine models and human patients with pancreatic tumors. Whether this represents a causal factor or a consequence of tumorigenesis remains unclear, emphasizing the need for further investigation into microbiome–mycobiome interactions [27]. Gut dysbiosis is frequently associated with increased intestinal permeability [28]. Under physiological conditions, bacteria and their products are contained and locally cleared by mucosal immune defenses. However, enhanced permeability allows translocation of bacterial components such as lipopolysaccharide (LPS)—a Gram-negative cell wall constituent that activates Toll-like receptor 4 (TLR4)—thereby inducing cytokine release, oxidative stress, and inflammation [28]. LPS has also been shown to promote tumor PD-L1 expression through activation of the NF-κB/AKT/MyD88 axis, enhancing resistance to T-cell-mediated cytotoxicity both in vitro and in vivo [29]. Microbial metabolites further modulate pancreatic oncogenesis through diverse mechanisms. Short-chain fatty acids (SCFAs)—including acetate, propionate, butyrate, and lactate—produced by bacterial fermentation of non-digestible carbohydrates, exert protective effects [30]. Acetate mitigates pancreatitis, thereby reducing a significant risk factor for tumorigenesis, while butyrate exhibits cytostatic properties in vitro by suppressing the proliferation of pancreatic adenocarcinoma cells [30]. Conversely, secondary bile acids display pro-tumorigenic activity. Microbial dehydroxylation and deconjugation of primary bile acids generate lithocholic acid (LCA), deoxycholic acid (DCA), and ursodeoxycholic acid (UDCA), a process mediated by Bacteroides, Firmicutes, and Lactobacillus species through their enzymatic repertoire [31,32]. Elevated levels of unconjugated bile acids have been reported in patients with pancreatic adenocarcinoma by O’Rees et al. [33]. Mechanistically, DCA binds the Takeda G protein-coupled receptor 5 (TGR5), activating the EGFR/STAT3 signaling pathway and promoting cell cycle progression [31]. In contrast, trimethylamine N-oxide (TMAO) has been associated with a reduced risk of pancreatic carcinogenesis. By activating the type I interferon (IFN) pathway, TMAO exerts anti-inflammatory effects and has been linked to improved survival in pancreatic cancer patients [34]. Another signaling pathway implicated in pancreatic oncogenesis is the mammalian target of rapamycin (mTOR) cascade, which regulates autophagy, cell growth, and cytoskeletal dynamics [35]. Collectively, these findings underscore the intricate interplay between the gut microbiota, microbial metabolites, and host signaling pathways in pancreatic carcinogenesis. They highlight the need for further mechanistic studies to clarify causality and to support the development of microbiome-targeted therapeutic strategies.

### 3.3. Intratumoral Microbiota in Pathogenesis and Progression of PDAC

Historically considered a sterile organ, the pancreas is now recognized to hold its own distinct microbiota [36]. Microbial species originating from the oral cavity, stomach, and intestine may colonize the common bile duct and subsequently migrate into the pancreatic duct [37]. The precise mechanisms by which microorganisms reach pancreatic tissue remain a matter of debate. One proposed pathway involves the translocation of duodenal microbes through the pancreatic ductal system, while alternative hypotheses suggest dissemination via mesenteric venous drainage or the lymphatic network [38,39]. Emerging evidence suggests that the intratumoral microbiota may play a role in pancreatic cancer development. Riquelme et al. analyzed microbiota directly isolated from resected pancreatic ductal adenocarcinoma (PDAC) tissue and demonstrated that patients with long-term survival (≥5 years after surgery) exhibited significantly higher alpha-diversity, as determined by 16S rRNA gene sequencing [40]. Similarly, beta-diversity analyses revealed an enrichment of Alphaproteobacteria, Sphingobacteria, and Flavobacteria in long-term survivors, whereas Clostridia and Bacteroides predominated in tumors from short-term survivors [40]. Further studies have suggested that the presence of specific microbial taxa within the tumor microenvironment—such as Enterobacter, Enterococcus, and Escherichia coli (*E. coli*) bactibilia—as well as chronic infection with hepatitis B or C viruses, may contribute to pancreatic carcinogenesis [41]. Nonetheless, it remains unclear whether these microbial communities act as active participants in tumor initiation and progression or merely represent secondary colonizers of already neoplastic tissue.

## 4. Gut Microbiota Influence on Chemotherapy and Immunotherapy

The gut microbiota has recently been identified as one of the “enabling characteristics” within the updated framework of the hallmarks of cancer [42]. A growing body of evidence from in vitro studies, animal models, and clinical investigations demonstrates that the microbiota contributes not only to cancer initiation and progression but also to the modulation of responses to chemotherapy and immunotherapy, thereby influencing mechanisms of therapeutic resistance [43,44]. Moreover, emerging data highlight its role in cancer therapy-related toxicities, further underscoring its potential significance as a therapeutic target.

### 4.1. Impact of the Microbiota on Therapeutic Efficacy in PDAC

Approximately 80% of pancreatic ductal adenocarcinoma (PDAC) cases are diagnosed at advanced stages, precluding surgical resection [45]. Consequently, chemotherapy remains the main treatment for most patients [46]. For unresectable PDAC, first-line regimens include combinations of 5-fluorouracil (5-FU), irinotecan, and oxaliplatin with folinic acid (FOLFIRINOX), or gemcitabine with nab-paclitaxel (GnP) [46,47]. However, PDAC is frequently characterized by both intrinsic and rapidly acquired resistance to chemotherapy [48], with fewer than half of patients exhibiting a favorable response [49]. This pronounced chemoresistance is attributed to several defining features of PDAC, including its complex genetic landscape, metabolic reprogramming, and the unique properties of its tumor microenvironment (TME) [50]. Hallmarks of the PDAC TME, such as a dense desmoplastic stroma composed of extracellular matrix (ECM), collagen, and activated pancreatic stellate cells, create a physical barrier that limits drug penetration, reduces tumor perfusion, and impedes immune cell infiltration [51,52]. Furthermore, the relatively high bacterial load within the PDAC TME compared with other malignancies underlines its susceptibility to microbial colonization [53], highlighting opportunities for microbiome-directed approaches to overcome drug resistance. Several studies have linked alterations in gut microbiota composition and specific microbial taxa to reduced cytotoxic efficacy of chemotherapy [43,44]. Geller et al. demonstrated that Mycoplasma hyorhinis within human dermal fibroblasts induced gemcitabine resistance in both in vitro and murine models [54]. Similarly, members of the Gammaproteobacteria can inactivate gemcitabine through the expression of a long isoform of cytidine deaminase (CDD), which converts the drug into its inactive metabolite [54]. In a cohort of 113 PDAC tissue samples, Gammaproteobacteria were detected in 76% of cases. In colorectal cancer murine models, the introduction of gemcitabine-degrading bacteria conferred resistance, which was reversed by co-administration of ciprofloxacin [54]. Consistent with these findings, a retrospective analysis by Weniger et al. reported that patients with PDAC receiving adjuvant gemcitabine had improved progression-free survival (PFS) in the absence of *Klebsiella pneumoniae* in bile cultures compared with those harboring the bacterium [55]. Additional evidence suggests that *Escherichia coli* (*E. coli*) contributes to chemoresistance. Lehouritis et al. demonstrated that a non-pathogenic *E. coli* strain altered the chemical structure of several chemotherapeutic agents, including gemcitabine, thereby inducing resistance in both in vitro and in vivo murine models [56]. In colorectal and pancreatic models, *Fusobacterium nucleatum* has been associated with 5-FU resistance through inhibition of apoptotic pathways via a TLR4/MYD88-dependent mechanism [57,58,59]. Conversely, certain commensal bacteria may enhance chemotherapy efficacy. Non-enterotoxigenic Bacteroides fragilis has been shown to stimulate T-cell-mediated immune responses that potentiate oxaliplatin-induced tumor apoptosis within the TME [60]. Likewise, butyrate-producing bacteria have been associated with improved oxaliplatin responsiveness through activation of CD8^+^ T cells, while antibiotic-mediated depletion of these species increased tumor resistance [60]. Panebianco et al. further demonstrated that butyrate enhanced the efficacy of gemcitabine in in vitro PDAC models, primarily by inducing apoptosis [61]. Beyond direct bacterial mechanisms, microbial metabolites also modulate the drug response [43,44]. Indole-3-acetic acid (3-IAA), a tryptophan-derived metabolite, was found at higher concentrations in patients who responded to chemotherapy [62]. This compound promotes the accumulation of reactive oxygen species (ROS), compromises the metabolic integrity of cancer cells, and enhances treatment efficacy. Strategies such as fecal microbiota transplantation, dietary interventions to increase tryptophan intake, and oral 3-IAA supplementation have been shown to improve chemotherapy outcomes in humanized gnotobiotic PDAC mouse models [62]. In addition to chemotherapy resistance, the gut microbiota has been implicated in modulating immunotherapy responses in PDAC. Although immune checkpoint inhibitors (ICIs) have demonstrated clinical benefit in several malignancies, fewer than 1% of PDAC patients respond [63]. This limited efficacy is attributed mainly to the immunosuppressive TME and the low tumor mutational burden characteristic of PDAC [64]. Significantly, microbiota-driven modulation of the TME may influence ICI responsiveness. Pushalkar et al. showed that antibiotic-mediated microbial ablation upregulated PD-1 expression and enhanced the antitumor effects of PD-1 blockade in a PDAC mouse model [38].

Collectively, these findings suggest that microbial communities and their metabolites play a role in both chemotherapy resistance and immune evasion in PDAC. Microbiota-targeted interventions, including dietary modification, prebiotics, probiotics, selective antibiotics, and fecal microbiota transplantation, represent promising strategies to enhance therapeutic efficacy (Table 1).

### 4.2. Impact of the Microbiota on Antitumor Therapy-Related Toxicities

As previously discussed, the gut microbiota plays a pivotal role in modulating the metabolism of chemotherapeutic drugs, thereby influencing both treatment efficacy and the host’s susceptibility to therapy-related toxicities [65]. Preclinical studies have shown that 5-fluorouracil (5-FU) increases the relative abundance of pathogenic taxa such as Escherichia, Enterococcus spp., and Clostridium, predisposing patients to mucositis, bacteremia, and sepsis [66,67]. Consistent with these findings, cisplatin-induced toxicity has been attenuated by the administration of D-methionine, which supports the growth of beneficial taxa, including Lachnospiraceae and Lactobacillus [66,67]. The gut microbiota also modulates irinotecan-induced toxicity. Irinotecan is converted into its active metabolite, SN-38, through hepatic glucuronidation and subsequently excreted into the intestinal lumen, where microbial β-glucuronidases hydrolyze it back to SN-38. This reactivated metabolite causes direct mucosal injury, leading to severe diarrhea that frequently requires dose reduction or treatment discontinuation [68,69,70]. Dysbiosis—whether induced by chemotherapy or by the disease itself—results in the overrepresentation of β-glucuronidase-rich taxa such as Proteobacteria, Clostridium clusters, and Fusobacteria, further exacerbating gastrointestinal toxicity and inflammation [71]. Given its pivotal role in chemotherapy-associated toxicities, modulation of the gut microbiota has emerged as a promising therapeutic strategy. Preclinical models have demonstrated that selective inhibition of microbial β-glucuronidases, combined with probiotic supplementation, markedly reduces SN-38 reactivation, thereby limiting mucosal injury, oxidative stress, and inflammatory cytokine release [71]. Panebianco et al. further reported that in vivo probiotic administration counteracted dysbiosis induced by gemcitabine plus nab-paclitaxel, decreased chemotherapy-related side effects and stromal activation, improved microbial diversity, and increased the abundance of SCFA-producing bacteria [72]. Despite these encouraging preclinical findings, rigorously designed randomized clinical trials are still required to confirm the safety and efficacy of microbiota-targeted interventions for mitigating treatment-related toxicities in pancreatic cancer (Table 2).

## 5. Microbiota Modulation in the Treatment of PDAC: Classic and New Therapeutic Strategies

The intestinal microbiota has emerged as a critical component in PDAC, shaping tumorigenesis, antitumor immunity, and therapeutic responsiveness. Multiple therapeutic strategies, including antibiotics, probiotics, dietary interventions, engineered bacteria, oncolytic viruses, and fecal microbiota transplantation (FMT), are currently under investigation to restore microbial homeostasis and improve treatment outcomes [21]. Increasingly, modulation of the microbiota is a key determinant of therapeutic efficacy in both chemotherapy and immunotherapy. In the context of immunotherapy, particularly with immune checkpoint inhibitors (ICIs) targeting PD-1/PD-L1 and CTLA-4, the gut microbiota modulates immune responses. It can regulate both systemic and intratumoral immunity by affecting the expression of checkpoint proteins and promoting remodeling of the tumor microenvironment. Through these mechanisms, the microbiota may enhance the efficacy of checkpoint blockade and improve response rates [73]. In chemotherapy, regimens like gemcitabine and FOLFIRINOX are challenged by desmoplastic stroma and multidrug resistance. Microbial communities can affect drug metabolism, bioactivation, and tissue penetration [74]. Targeted manipulation of the gut, oral, or intratumoral microbiota is a promising strategy for overcoming therapeutic resistance and enhancing the effects of established cytotoxic and immune-based treatments. This approach could open new avenues to improve clinical outcomes in patients with pancreatic ductal adenocarcinoma (PDAC).

### 5.1. Antibiotics

Antibiotic therapy has shown promising results in altering the microbiota and improving the response to chemotherapy, particularly gemcitabine. In preclinical models, administration of broad-spectrum antibiotics reduced tumor burden by depleting microbial populations [75]. Two independent studies further demonstrated that antibiotic-mediated microbiota ablation reprograms antitumor immune responses and influences PDAC progression. Sethi et al. reported that antibiotics delayed tumor growth in subcutaneous PDAC models by promoting the infiltration of IFN-γ-producing cytotoxic T cells, while suppressing immunosuppressive T cells that secrete IL-17A and IL-10 [76]. Similarly, Pushalkar et al. observed that microbial ablation in orthotopic PDAC models decreased tumor burden and induced an immunostimulatory microenvironment, characterized by increased M1 macrophages, CD4^+^ Th1 cells, and CD8^+^ T cells, alongside a reduction in MDSCs and TLR2/5 signaling [38]. This immune reprogramming may also enhance chemotherapy efficacy, as intratumoral Gammaproteobacteria can express the long isoform of cytidine deaminase (CDD), which inactivates gemcitabine and promotes resistance [54,77]. Elevated intratumoral bacterial loads have been correlated with reduced gemcitabine efficacy, an effect reversed following postoperative antibiotic therapy [78,79]. Clinical data corroborate these preclinical findings. Patients receiving antibiotics during gemcitabine–nab-paclitaxel (GnP) treatment exhibited longer progression-free survival (5.8 vs. 2.7 months) and improved overall outcomes [80]. Retrospective analyses further support the benefit of concurrent antibiotic therapy, demonstrating improved overall survival (OS) and progression-free survival (PFS) when combined with gemcitabine, but not with fluoropyrimidines, suggesting a drug-specific interaction with the microbiota [81]. In a cohort of 216 patients, first-line gemcitabine plus antibiotics significantly prolonged both OS and PFS, whereas fluoropyrimidine-based regimens yielded only marginal PFS benefits [80]. Among antibiotic classes, specific agents appear more effective. Ciprofloxacin has been shown to counteract gemcitabine resistance mediated by Gammaproteobacteria [54]. Both macrolides and quinolones, when used in combination with chemotherapy, are associated with survival gains of approximately 2–3 months in metastatic PDAC [80]. Quinolones are particularly effective in patients colonized with Klebsiella pneumoniae [55], while macrolide therapy lasting more than three days has been independently correlated with improved OS and PFS [80]. Despite these encouraging findings, concerns remain regarding prolonged use of broad-spectrum antibiotics. Documented risks include gastrointestinal, hepatic, and hematologic toxicities [82]; dysbiosis and opportunistic infections [83]; and the emergence of antimicrobial resistance. To mitigate systemic toxicity, localized antibiotic delivery systems have been explored. In a murine colon carcinoma model, microdevices implanted with ampicillin and chloramphenicol directly into tumors inhibited growth and enhanced apoptosis. When combined with gemcitabine, these effects were further amplified, underscoring the critical role of intratumoral bacteria in mediating chemoresistance [54] (Table 3).

### 5.2. Probiotics and Prebiotics

Probiotics have gained increasing attention as non-invasive strategies to modulate microbial communities. Because microbes from the oral–gut axis may translocate to the pancreas via the pancreatic duct, oral probiotic administration represents a plausible route of intervention, although clinical validation remains necessary [84]. *Lactobacillus* species—including *L. paracasei, L. reuteri,* and *L. rhamnosus*—exhibit antitumor activity by inhibiting tumor growth and metastasis while enhancing the efficacy of gemcitabine. These effects involve p53 activation, cell cycle arrest, and modulation of epithelial–mesenchymal transition (EMT) markers [83,85,86]. In murine models, co-administration of *Lactobacillus spp* with gemcitabine significantly attenuated PanIN progression and improved hepatic enzyme profiles, suggesting both enhanced tolerability and therapeutic efficacy [86]. Additional studies have shown that *L. reuteri* suppresses PDAC progression by promoting natural killer (NK) cell infiltration, and higher fecal abundance of *Lactobacillus* correlates with improved survival [87]. Beyond probiotics, postbiotic compounds derived from these microorganisms also display therapeutic potential. Ferrichrome, secreted by *L. casei*, exerts antineoplastic effects even in 5-FU-resistant cell lines via p53-mediated apoptotic pathways [88]. Similarly, *Bifidobacterium longum* attenuates PDAC progression by enhancing CD8^+^ T-cell infiltration and reducing immunosuppressive cell subsets [89]. This species also synergizes with anti-PD-L1 therapy through dendritic cell activation [90]. Notably, when combined with vancomycin and neomycin, *B. longum* further improved antitumor responses and promoted CD8^+^ T-cell recruitment in murine models [91]. Another relevant species, *Akkermansia muciniphila*, has been shown to enhance CD8^+^ T-cell-mediated immunity and improve responses to immune checkpoint inhibitors [92,93]. Further evidence supports the efficacy of synbiotics—combinations of probiotics and prebiotics—in optimizing host–microbe interactions. In a randomized trial involving 90 PDAC patients, perioperative administration of probiotics plus inulin increased intratumoral CD8^+^ T-cell infiltration and IFN-γ expression, as demonstrated by immunohistochemistry of resected tumors [91]. Synbiotic therapy also reduced circulating levels of pro-inflammatory cytokines (IL-1β, IL-6, and IL-10) [91]. Clinically, these effects were associated with fewer postoperative complications and a lower incidence of bacteremia, underscoring the potential role of synbiotics in perioperative management [94]. Postbiotics—defined as bioactive microbial-derived metabolites—represent another promising frontier in microbiota-based therapy. SCFA, particularly butyrate, together with metabolites such as indole-3-acetic acid, exert regulatory effects on both tumor and immune cells. Butyrate, produced through microbial fermentation of dietary fibers, enhances gemcitabine efficacy by inducing oxidative stress and autophagy [62]. It also reduces tumor proliferation by inhibiting histone deacetylase (HDAC) and modulating epigenetics, highlighting its multifaceted role in pancreatic cancer therapy [95] (Table 4).

### 5.3. Engineered Bacteria

The potential of engineered bacteria as ‘living drugs’ to directly target the tumor microenvironment is a source of great optimism. For instance, hypoxia-responsive *Escherichia coli* strains have been genetically modified to secrete cyst(e)inase (CGL), leading to the depletion of intracellular cysteine and cystine pools. This process induces ferroptosis through lipid peroxidation while also activating antitumor immune responses [96]. Other approaches involve the use of synthetic *Salmonella* strains engineered to deliver STING agonists via outer membrane vesicles (OMVs). These bacterial strains enhance immune cell infiltration and act synergistically with immune checkpoint inhibitors [97]. Similarly, attenuated *Salmonella Typhimurium* strains engineered to secrete immunomodulatory proteins have been shown to inhibit PDAC growth and modulate cytokine profiles [98]. Another innovative strategy involves the functionalization of *Lactobacillus rhamnosus* GG with a gallium–polyphenol network (LGG@Ga-poly). This formulation effectively depletes Proteobacteria and lipopolysaccharides (LPSs), remodels the tumor immune microenvironment, and enhances the efficacy of immunotherapy [99] (Table 5).

### 5.4. Non-Bacterial Microbiome Components: Bacteriophages and Oncolytic Viruses

Lytic bacteriophages provide a highly selective method for eliminating pathogenic bacteria associated with tumors. Additionally, phage display technologies have enabled the identification of tumor-specific peptides, such as MCA1, which bind to PDAC cells with high specificity and nanomolar affinity. This presents potential applications for imaging and targeted drug delivery [100]. Fusion proteins derived from bacteriophages have also been employed to functionalize liposomes containing doxorubicin, thereby enhancing tumor selectivity and cytotoxic potency [101]. Similarly, polymeric micelles functionalized with phage-derived proteins and loaded with paclitaxel have demonstrated improved tumor targeting, increased drug solubility, and reduced off-target toxicity [102]. Oncolytic viruses (OVs) represent a distinct class of biotherapeutics capable of interacting with the microbiota and remodeling the tumor microenvironment. Adenoviruses have been extensively investigated due to their stability and high transduction efficiency. While first-generation vectors such as ONYX-015 exhibited limited replication capacity and were rapidly neutralized by host immunity [103,104], next-generation adenoviral platforms have been engineered to overcome antiviral resistance [105], enhance tumor radiosensitivity [106], and modulate stromal components [107]. Herpes simplex virus (HSV)-based OVs have been shown to induce apoptosis and necrosis of tumor cells in preclinical PDAC models while synergizing with gemcitabine [108]. Naturally mutated HSV strains have further demonstrated the ability to activate CD4^+^/CD8^+^ T cells and NK cells in early-phase clinical trials [109]. Vaccinia virus strains have also exhibited antitumor effects, either as monotherapy or in combination with chemotherapy and radiotherapy, with encouraging preclinical outcomes and ongoing clinical evaluation [110,111,112]. Reoviruses, which selectively replicate in RAS-mutant cells, are particularly well suited for PDAC. Pelareorep, a clinically tested reovirus, has shown limited efficacy as a single agent, but ongoing studies are exploring its synergistic potential with immune checkpoint inhibitors [113]. Similarly, the Coxsackievirus B3 strain PD-H has demonstrated potent lytic activity in vitro and significant tumor suppression in vivo, although its optimal efficacy may depend on combination strategies targeting the stromal compartment [114] (Table 6).

### 5.5. Fecal Microbiota Transplantation

Fecal microbiota transplantation (FMT) is rapidly emerging as a promising strategy to modulate both intestinal and tumor-associated microbiota in PDAC. Preclinical studies consistently demonstrate that FMT derived from immunotherapy responders or long-term PDAC survivors reduces tumor burden and enhances T-cell infiltration [75]. Riquelme et al. reported that long-term survivors exhibited a more diverse intratumoral microbiota, including Pseudoxanthomonas, Streptomyces, Saccharopolyspora, and Bacillus clausii (B. clausii), which correlated with improved clinical outcomes [40]. Similarly, Tintelnot et al. found that FMT from chemotherapy responders increased therapeutic sensitivity in murine models, whereas FMT from healthy donors inhibited tumor progression [62]. Growing evidence supports FMT as a valuable adjunct to immunotherapy [115]. By reshaping the gut microbiota, FMT has been shown to restore responsiveness to immune checkpoint inhibitors (ICIs) and to reduce the incidence and severity of immune-related adverse events (irAEs). Although PDAC-specific clinical trials are still lacking, studies in other malignancies provide proof of principle. In melanoma, FMT from responder patients reversed resistance to anti-PD-1/PD-1/PD-L1 therapy, and similar effects were observed using healthy donor-derived material [116,117,118]. In one clinical trial, three of fifteen anti-PD-1-refractory melanoma patients responded to FMT combined with pembrolizumab [119]. In another Phase I trial, FMT followed by anti-PD-1 reinduction achieved one complete and two partial responses among ten patients [116]. Nevertheless, safety concerns persist. A notable adverse event involved transmission of an ESBL-producing Escherichia coli strain from a shared donor, resulting in bacteremia and one fatality [120]. Despite this, FMT has shown clinical benefit in managing ICI-induced colitis. Several reports document its efficacy in steroid- and biologic-refractory cases, allowing continuation of cancer immunotherapy and potentially improving adherence to treatment protocols [121,122,123,124]. FMT may also mitigate radiotherapy-associated toxicity. In patients with refractory radiation enteritis, FMT led to clinical improvement without serious adverse events [125]. In a larger cohort, it was associated with improved gastrointestinal quality of life and nutritional status over a six-month follow-up [126]. In summary, although PDAC-specific trials remain limited, the rationale for microbiota-based interventions is compelling. Integration of FMT with chemotherapy, radiotherapy, or immunotherapy may enhance efficacy while reducing toxicity, particularly in neoadjuvant and perioperative settings. Further clinical studies are urgently needed to validate the safety, efficacy, and long-term benefits of this approach in PDAC management. (Table 7)

### 5.6. Future Direction

Several critical questions remain unresolved and are now the focus of ongoing clinical investigations. These include the long-term effects and safety profile of fecal microbiota transplantation (FMT) in oncology patients; the identification of specific microbial signatures associated with clinical features, molecular subtypes, or prognosis in PDAC; and the extent to which microbiota composition, microbial metabolites, and immune modulation influence disease progression and therapeutic response. To address these questions, multiple clinical trials are currently underway. The Microbiome of Pancreatic Cancer: “PANDEMIC study” (NCT04274972) is prospectively evaluating whether distinct gut microbial patterns correlate with disease characteristics and outcomes. The PDA-MAPS trial (NCT04922515) is conducting an integrated analysis of oral, gut, and tumor-associated microbiota, combined with genomic data, to determine whether microbiome-based subtypes of PDAC can be defined. Metabolomic and Immune–Microbiome Profiling for Unresectable Pancreatic Cancer (NCT07036978) aims to elucidate how microbial metabolites and host immune responses modulate tumor behavior in advanced disease. In addition, an early-phase study, “Fecal Microbial Transplants for the Treatment of Pancreatic Cancer” (NCT04975217), is assessing the safety and immunomodulatory potential of FMT in patients undergoing preoperative management. In contrast, issues related to the standardization, scalability, and biosafety of engineered bacterial- and viral-based therapies remain largely unaddressed by current clinical trials. Collectively, these efforts underscore a growing recognition that the microbiome may provide critical insights—and potentially novel therapeutic avenues—for improving outcomes in pancreatic cancer.

## 6. Conclusions

The microbiota is increasingly recognized not only as a driver of PDAC pathogenesis but also as a potent modulator of therapeutic response. From the oral axis to the tumor microenvironment, distinct microbial taxa and their metabolites can either suppress cytotoxic efficacy or, conversely, enhance immune surveillance. Preclinical studies have demonstrated that antibiotics, probiotics, engineered bacteria, bacteriophages, oncolytic viruses, and fecal microbiota transplantation can remodel the desmoplastic and immunosuppressive tumor niche, restoring sensitivity to both chemotherapy and immunotherapy. These findings delineate a new therapeutic frontier. Microbiota modulation—through antibiotics, probiotics, synbiotics, postbiotics, engineered “living drugs,” bacteriophages, oncolytic viruses, or fecal transplantation—holds the potential to overcome chemoresistance, restore immunogenicity, and mitigate treatment-related toxicity. Early clinical evidence, particularly with synbiotic therapy and responder-derived FMT, indicates promising translational potential. However, rigorous clinical validation remains essential. Well-designed, adequately powered trials are necessary to define the efficacy, safety, and optimal integration of microbiota-based approaches into existing treatment regimens. Ultimately, harnessing the microbiome may help transform one of oncology’s most treatment-refractory malignancies into a disease amenable to precision immunotherapy.

## Figures and Tables

**Table 1:** Microbiota and Therapy in PDAC.

Author/Study	Microorganism/Metabolite	Drug/Therapy	Mechanism	Effect on Therapy
Geller et al. 2017 [54]	*M. hyorhinis*; Gammaproteobacteria	Gemcitabine	Gemcitabine inactivation via cytidine deaminase (CDD)	Resistance
Weniger et al. 2021 [55]	*K. pneumoniae*	Gemcitabine	Presence associated with reduced PFS under gemcitabine	Resistance
Lehouritis et al. 2015 [56]	*E. coli* (non-pathogenic strain)	Gemcitabine and others	Drug structural modification, leading to resistance	Resistance
Ye et al., Udayasuryan et al., Yu et al. 2017, 2022, 2024 [57,58,59]	*F. nucleatum*	5-FU	TLR4/MYD88-dependent inhibition of apoptosis	Resistance
Bai et al. 2023 [60]	*B. fragilis* (non-toxigenic)	Oxaliplatin	T-cell activation enhances oxaliplatin-induced apoptosis	Sensitization
Bai et al., 2023 Panebianco et al. 2022 [60,61]	Butyrate-producing bacteria	Oxaliplatin	CD8+ T-cell activation; depletion increases resistance	Sensitization
Panebianco et al. 2022 [61]	Butyrate	Gemcitabine	Induction of apoptosis in PDAC cells	Sensitization
Tintelnot et al. 2023 [62]	Indole-3-acetic acid (3-IAA)	Gemcitabine	ROS accumulation, impaired cancer metabolism	Sensitization
Pushalkar et al. 2018 [38]	Multiple species (antibiotic-sensitive)	Anti-PD-1 immunotherapy	Microbial ablation enhances PD-1 blockade efficacy	Sensitization

CDD: Cytidine deaminase, PFS: Progression-free survival, TLR4: Toll-like receptor 4, MYD88: Myeloid differentiation primary response 88, PDAC: Pancreatic ductal adenocarcinoma, ROS: Reactive oxygen species, PD-1: Programmed cell death protein 1.

**Table 2:** Gut Microbiota and Chemotherapy-Related Toxicities in PDAC.

Drug	Microorganism/Metabolite	Toxicity	Mechanism	Intervention/Findings
5-Fluorouracil	*Escherichia, Enterococcus spp., Clostridium*	Mucositis, bacteremia, sepsis	Pathogenic overgrowth predisposes to infections	Association with dysbiosis 2017, 2018 [66,67]
Cisplatin	Lachnospiraceae, *Lactobacillus* (protective, promoted by D-methionine)	General toxicity (attenuated by microbiota modulation)	D-methionine fosters beneficial taxa, reducing toxicity	D-methionine promotes protective taxa 2017, 2018 [66,67]
Irinotecan	*Proteobacteria, Clostridium clusters*, *Fusobacteria* (β-glucuronidase producers)	Severe diarrhea, mucosal injury	β-glucuronidase hydrolyzes SN-38G back to SN-38, damaging mucosa	Targeted GUS inhibitors and probiotics reduce toxicity 2020, 2019, 2021, 2023 [68,69,70,71]
Gemcitabine + Nab-paclitaxel	SCFA-producing bacteria (butyrate producers)	Dysbiosis, chemotherapy side effects, stromatogenesis	Loss of SCFA producers; restored with probiotics, improving diversity	Probiotic supplementation mitigates side effects, restores SCFA producers 2023 [72]

GUS: β-glucuronidase, SN-38: Active irinotecan metabolite, SN-38G: SN-38 glucuronide, SCFA: Short-chain fatty acid.

**Table 3:** Antibiotics and Microbiota Modulation in PDAC Therapy.

Study/Author	Model/Setting	Antibiotic/Intervention	Mechanism/Finding	Outcome
Sethi et al. 2018 [76]	Subcutaneous PDAC (murine)	Broad-spectrum antibiotics	↑ IFN-γ cytotoxic T cells; ↓ IL-17A/IL-10 T cells	Delayed tumor growth (preclinical)
Pushalkar et al. 2018 [38]	Orthotopic PDAC (murine)	Broad-spectrum antibiotics	↑ M1 macrophages, CD4+ Th1, CD8+; ↓ TLR2/5 signaling	Reduced tumor burden (preclinical)
Weniger et al. 2021[55]	Preclinical/human tissue	Ciprofloxacin (counteracts Gammaproteobacteria)	Reverses Gammaproteobacteria-mediated gemcitabine resistance	Restored gemcitabine efficacy
Fulop et al. 2023 [81]	Clinical cohort, gemcitabine+nab-paclitaxel	Antibiotics during GnP treatment	Improved PFS (5.8 vs 2.7 months); better outcomes	Clinical survival benefit
Weniger et al. 2021 [55]	Clinical cohort, bile culture (*K. pneumoniae*)	Absence of Klebsiella pneumoniae	Better PFS when *K. pneumoniae* is absent	Improved progression-free survival
Hajishengallis et al. 2012 [82]	Clinical retrospective cohort	Antibiotics + Gemcitabine vs Fluoropyrimidines	Gemcitabine benefits, not with fluoropyrimidines	Improved OS/PFS with gemcitabine
Fulop et al. 2023 [81]	Clinical metastatic PDAC	Macrolides and quinolones	Survival benefit of +2–3 months	Improved OS/PFS in metastatic PDAC
Geller et al. 2017 [54]	Murine colon carcinoma model	Local release of ampicillin, chloramphenicol	Tumor inhibition, apoptosis ↑; synergy with gemcitabine	Reduced chemoresistance; local effect with less systemic toxicity

PDAC: Pancreatic ductal adenocarcinoma, IFN: Interferon, IL: Interleukin, CD: Cluster of differentiation, Th: T helper, TLR2/5: Toll-like receptor 2/5, GnP: Gemcitabine/nab-paclitaxel, PFS: Progression-free survival, OS: Overall survival.

**Table 4:** Probiotics, Synbiotics, and Postbiotics in PDAC.

Species/Molecule	Mechanism	Therapeutic Effect	Model/Setting
*Lactobacillus paracasei*, *L. reuteri*, *L. rhamnosus*	p53 activation, cell cycle arrest, EMT modulation	Antitumor activity; enhances gemcitabine efficacy	Preclinical models 2005, 2020 [83,85,86]
*Lactobacillus* (co-administered with gemcitabine)	Reduced PanIN progression; improved hepatic enzyme profile	Improved drug tolerability and efficacy	Mouse models 2020 [86]
*Lactobacillus reuteri*	Promotes NK cell infiltration; correlated with improved survival	Suppression of PDAC progression; survival benefit	Mouse and clinical correlations 2025 [87]
Ferrichrome (from *L. casei*)	p53-mediated apoptosis, including in 5-FU-resistant cells	Antineoplastic effect	In vitro models 2020 [88]
*Bifidobacterium longum*	Enhances CD8^+^ T-cell infiltration; reduces immunosuppressive subsets; synergizes with anti-PD-L1	Attenuates PDAC progression; potentiates immunotherapy	Preclinical models 2020, 2015, 2024 [89,90,91]
*Akkermansia muciniphila*	Boosts CD8^+^ T-cell immunity; enhances checkpoint inhibitor efficacy	Improved immunotherapy response	Preclinical and translational 2023, 2024 [92,93]
Synbiotics (probiotics + inulin)	↑ CD8^+^ T-cell infiltration and IFN-γ; ↓ IL-1β, IL-6, IL-10	Reduced postoperative complications and bacteremia	Randomized clinical trial (90 patients) 2024, 2023 [91,94]
Butyrate (SCFA)	Enhances gemcitabine efficacy (oxidative stress, autophagy); HDAC inhibition; epigenetic regulation	Suppresses tumor proliferation; potentiates chemotherapy	Preclinical and mechanistic studies 2023 [62,95]
Indole-3-acetic acid	Regulates tumor and immune cell activity; enhances chemotherapy response	Enhanced chemotherapy effectiveness	Preclinical models 2023 [62]

EMT: Epithelial–mesenchymal transition, PanIN: Pancreatic intraepithelial neoplasia, NK: Natural killer, FU: Fluorouracil, CD: Cluster of differentiation, PD-L1: Programmed death-ligand 1, IFN: Interferon, IL: Interleukin, SCFA: Short-chain fatty acid, HDAC: Histone deacetylase.

**Table 5:** Engineered Bacteria as Therapeutic Tools in PDAC.

Engineered Bacterium	Strategy	Mechanism	Therapeutic Effect
Hypoxia-responsive *E. coli*	Genetically modified to secrete cyst(e)inase (CGL)	Depletes cysteine/cystine pools → induces ferroptosis via lipid peroxidation; activates antitumor immunity	Ferroptosis induction and immune activation 2025 [96]
Synthetic *Salmonella* strains (OMVs with STING agonists)	Delivery of STING agonists via outer membrane vesicles	Enhances immune infiltration; synergizes with checkpoint inhibitors	Improved immunotherapy efficacy 2024 [97]
Attenuated *Salmonella Typhimurium* (immunomodulatory proteins)	Engineered to secrete immunomodulatory proteins	Inhibits PDAC growth; alters cytokine profiles	Suppression of PDAC growth 2022 [98]
*Lactobacillus rhamnosus GG* functionalized with Ga–polyphenol network (LGG@Ga-poly)	Functionalization with gallium–polyphenol network	Depletes Proteobacteria and LPS; remodels tumor immune microenvironment	Enhanced immunotherapeutic response 2024 [99]

CGL: Cystathionine γ-lyase, OMVs: Outer membrane vesicles, STING: Stimulator of interferon genes, PDAC: Pancreatic ductal adenocarcinoma, Ga–polyphenol: Gallium–polyphenol complex, LPS: Lipopolysaccharide.

**Table 6:** Bacteriophages and Oncolytic Viruses in PDAC.

Agent	Strategy	Mechanism	Therapeutic Effect
Lytic bacteriophages	Eradication of tumor-associated pathogenic bacteria	Selective bacterial lysis	Potential elimination of pathogenic microbiota 2020 [100]
Phage peptide MCA1	Phage display-identified peptide binds PDAC cells	High-specificity binding with nanomolar affinity	Imaging and targeted drug delivery 2020 [100]
Phage-decorated liposomes (doxorubicin)	Fusion proteins used to decorate liposomes	Enhanced tumor selectivity and cytotoxicity	Enhanced doxorubicin efficacy 2014 [101]
Phage-functionalized micelles (paclitaxel)	Micelles functionalized with phage proteins	Improved drug targeting and solubility; reduced off-target toxicity	Improved paclitaxel delivery and safety, 2018 [102]
Adenoviruses (first-generation ONYX-015)	First oncolytic adenovirus vector	Limited replication; immune clearance	Limited efficacy 2018. 2017 [103,104]
Next-generation adenoviruses	Engineered to overcome antiviral resistance, radiosensitize, and remodel stroma	Enhanced persistence, radiosensitivity, and stromal modulation	Improved antitumor activity 2018. 2007 [105,106,107]
HSV-based oncolytic viruses	Engineered or naturally mutated strains	Induces apoptosis/necrosis; stimulates immune activation	Suppressed PDAC growth; synergy with gemcitabine 2015, 2017 [108,109]
Vaccinia virus strains	Engineered or naturally attenuated strains	Direct oncolysis; synergizes with chemo/radiotherapy	Preclinical efficacy; ongoing clinical trials 2009. 2014, 2015 [110,111,112]
Reoviruses (Pelareorep)	Exploits replication in RAS-mutant cells	Replicates in RAS-mutant cells; immunotherapy synergy	Limited monotherapy efficacy; synergy with ICIs 2020 [113]
Coxsackievirus B3 (PD-H strain)	Naturally lytic strain targeting PDAC	Potent lytic activity; requires stromal-targeting combination	Suppressed tumor growth in vivo 2024 [114]

MCA1: Mia PaCa-2 Cell-binding peptide 1, PDAC: Pancreatic ductal adenocarcinoma, HSV: Herpes simplex virus, RAS: Rat sarcoma virus, ICIs: Immune checkpoint inhibitors.

**Table 7:** Fecal Microbiota Transplantation in PDAC.

Study/Setting	Donor/Source	Outcome
Chandra et al. 2021 [75]	Immunotherapy responders or long-term PDAC survivors	Reduced tumor burden; ↑ T-cell infiltration
Riquelme et al. 2019 [40]	Long-term PDAC survivors (diverse intratumoral microbiota)	Higher intratumoral diversity correlated with survival (*Pseudoxanthomonas, Streptomyces, Saccharopolyspora, B. clausii*)
Tintelnot et al. 2023 [62]	Chemotherapy responders or healthy donors	Improved chemosensitivity; reduced tumor progression
No Melanoma trials (anti-PD-1 refractory) 2021, 2023, 2024 [116,117,118,119]	Responder or healthy donors	Reversal of anti-PD-1 resistance; partial and complete responses
De Filipp et al. 2019 [120]	Shared donor (safety incident)	Fatal bacteremia due to ESBL-producing *E. coli*
ICI-induced colitis (steroid/biologic-refractory) 2023, 2024, 2018, 2022 [121,122,123,124]	Responder-derived or healthy donor FMT	Improved colitis control; enabled ICI continuation
Ding et al. 2020 [125]	FMT donors (clinical setting)	Clinical improvement without serious adverse events
Cui et al. 2023 [126]	FMT donors (extended follow-up)	Improved GI quality of life and nutritional status (6 months)

PDAC: Pancreatic ductal adenocarcinoma, PD-1: Programmed cell death protein 1, ESBL: Extended-spectrum beta-lactamases, ICI: Immune checkpoint inhibitor, FMT: Fecal microbiota transplantation, GI: Gastrointestinal.

## Data Availability

Not applicable.

## Abbreviations

The following abbreviations are used in this manuscript:

PDAC	Pancreatic ductal adenocarcinoma
MeSH	Medical subject headings
SNP	Single-nucleotide polymorphism
LPS	Lipopolysaccharide
TLR4	Toll-like receptor 4
MyD88	Myeloid differentiation primary response 88
PD-L1	Programmed death-ligand 1
NF-κB	Nuclear factor kappa-light-chain-enhancer of activated B cells
AKT	Protein kinase B
SCFAs	Short-chain fatty acids
LCA	Lithocholic acid
DCA	Deoxycholic acid
UDCA	Ursodeoxycholic acid
TGR5	Takeda G protein-coupled receptor 5
EGFR	Epidermal growth factor receptor
STAT3	Signal transducer and activator of transcription 3
TMAO	Trimethylamine N-oxide
IFN	Interferon
mTOR	Mammalian target of rapamycin
ECM	Extracellular matrix
TME	Tumor microenvironment
5-FU	5-Fluorouracil
GnP	Gemcitabine plus nab-paclitaxel
PFS	Progression-free survival
OS	Overall survival
CDD	Cytidine deaminase
TLR2	Toll-like receptor 2
TLR5	Toll-like receptor 5
MDSCs	Myeloid-derived suppressor cells
ROS	Reactive oxygen species
3-IAA	Indole-3-acetic acid
ICI/ICIs	Immune checkpoint inhibitor(s)
PD-1	Programmed cell death protein 1
CD8^+^	Cytotoxic T lymphocyte (CD8 positive)
CD4^+^	Helper T lymphocyte (CD4 positive)
Th1	T helper 1 cell
M1	M1 macrophage (pro-inflammatory phenotype)
IL-1β	Interleukin 1 beta
IL-6	Interleukin 6
IL-10	Interleukin 10
IL-17A	Interleukin 17A
FOLFIRINOX	5-FU, leucovorin, irinotecan, oxaliplatin
GUS	β-glucuronidase
SN-38/SN-38G	7-ethyl-10-hydroxycamptothecin/SN-38 glucuronide
EMT	Epithelial–mesenchymal transition
NK	Natural killer cell
FMT	Fecal microbiota transplantation
STING	Stimulator of interferon genes
OMV/OMVs	Outer membrane vesicle(s)
OV/OVs	Oncolytic virus(es)
HSV	Herpes simplex virus
ONYX-015	First-generation oncolytic adenovirus vector
LGG	*Lactobacillus rhamnosus* GG
LGG@Ga-poly	LGG functionalized with gallium–polyphenol network
CGL	Cyst(e)inase enzyme
RAS	Rat sarcoma viral oncogene family
KRAS^G12D	KRAS gene mutation (glycine 12 to aspartate)
PanIN	Pancreatic intraepithelial neoplasia
KPC	*Kras*^G12D; *Trp53*^R172H; Pdx1-Cre mouse model
ATCC	American Type Culture Collection
MCA1	Phage-derived tumor-targeting peptide
irAEs	Immune-related adverse events
GI	Gastrointestinal
